# The Impact of Obesity and Exercise on Cognitive Aging

**DOI:** 10.3389/fnagi.2013.00097

**Published:** 2013-12-20

**Authors:** John S. Y. Chan, Jin H. Yan, V. Gregory Payne

**Affiliations:** ^1^Department of Psychology, The Chinese University of Hong Kong, Hong Kong, China; ^2^Institute of Affective and Social Neuroscience, Shenzhen University, Shenzhen, China; ^3^Department of Psychology, Tsinghua University, Beijing, China; ^4^Department of Kinesiology, San Jose State University, San Jose, CA, USA

**Keywords:** aging, exercise, functionality, neural mechanisms, overweight, well-being

## Abstract

Obesity is a major concern in the aging population and degrades health, motor functions and cognition in older adults. The effects of obesity are pervasive and challenging to health-care systems, making this a widespread and critically important public health dilemma. In this review, we examine the relationship between obesity, cognitive aging, and related dysfunctions. Potential neural mechanisms underlying such relationship are described. We propose that cost-effective exercises can be employed to cope with obesity and cognitive declines in older adults. Finally, we discuss implications and future research directions.

## Introduction

Obesity is an over-accumulation of body fat that adversely affects physical and mental health. A series of investigations confirms the associations between obesity, premature mortality, metabolic syndromes, cardiovascular diseases, and mobility-related disorders (Villareal et al., 2005; DeCaria et al., 2012). A range of measures are available for assessing obesity including body mass index (BMI = height in meters/weight in kilograms squared), a widely used indicator. A person with a BMI ≥30 is obese, but such determinations vary from one region to another (World Health Organization, 2013).

There has been an increase in adult obesity in the United States. Signs of acceleration of obesity have been noted in most countries (Stevens et al., 2012). In the US, the prevalence of obesity rises from 20 to 60 years of age, and decreases thereafter (Flegal et al., 1998; Mokdad et al., 2001). Longitudinal studies show that from 20 to 80 years of ages, fat mass and percentage of body fat assessed by hydrostatic weighing or skin-fold measurements, generally increase while fat-free mass decreases. Cross-sectional studies indicate that BMI increases from 20 to 60 years of ages and declines mildly thereafter relative to younger adults (Jackson et al., 2012). Though obesity may seem less severe after the age of 60, the obesity issue in older adults becomes an increasingly serious health issue considering the dramatic increase in number of obese older adults (Howel, 2012). The increasing severity in the US is a result of the prevalence of obesity among 65- to 74-year-old men increasing from 10.4% in 1960–1962 to 41.5% in 2007–2010. In women, the prevalence increased from 23.2 to 40.3% during the same period (U.S. Department of Health and Human Services, 2012).

In addition to impacting physical function in older adulthood, there is increasing evidence showing that obesity contributes to cognitive aging and dysfunctions. Challenges regarding older adults and obesity are multi-faceted and significant (Rössner, 2001), requiring cost-effective and administratively feasible solutions. In this article, we describe the effects of obesity on cognitive aging along with some possible underlying mechanisms. We also discuss exercise and its role in decreasing obesity and improving cognition in older adults. Finally, we posit and discuss implications and recommend future research regarding older adults, obesity, and cognition.

## Obesity, Cognitive Aging, and Related Dysfunctions

Strong connections exist between obesity and physical health (Nelson et al., 2007). A growing body of research indicates that obesity is also related to cognition, a collection of mental abilities including reception and processing of environmental information, and behavioral output related to that processing. The relation between obesity and cognition is reflected by the negative correlations between BMI and gray matter ratio in men, metabolic activity in prefrontal areas and the anterior cingulate cortex (ACC). ACC and prefrontal metabolic activity are closely linked to executive functions for goal-directed behaviors, like the ability to follow a plan flexibly (Taki et al., 2008; Volkow et al., 2008).

As one approaches older adulthood, structural and functional changes occur in the brain. This gives rise to declines in an array of cognitive abilities through a process commonly referred to as cognitive aging (Salthouse, 2004). Global cognitive measures (e.g., Mini Mental State Examination) and intelligence tests often show higher scores in normal than obese individuals (Elias et al., 2005; Jeong et al., 2005; Kerwin et al., 2010). Though age-related declines are observed in global cognitive measures, specific cognitive abilities (e.g., executive functions), are particularly vulnerable in older adults.

The association between obesity and cognitive aging is especially high in midlife and weaker in late life. However, the effect of obesity on cognition of older adults should not be overlooked or underestimated (Dahl and Hassing, 2013). Studies show that obesity is correlated with anatomical and functional changes in the aging brain. BMI is positively related to smaller brain volume in obese older adults. Compared to those with normal BMI, obese older adults show atrophy in the frontal lobes, ACC, hippocampus, and thalamus (Raji et al., 2010). There are inverse relationships between obesity indices (BMI, abdominal girth) and cerebral white matter integrity (Marks et al., 2011). In older adults with Alzheimer’s disease (AD), or mild cognitive impairments (MCI), higher BMI is related to volumetric reductions in frontal, temporal, parietal, and occipital lobes (Ho et al., 2010). Waist-to-hip ratio, a measure of obesity, is also negatively correlated with hippocampal volume (Jagust et al., 2005).

In the rest of this section, we discuss the effects of obesity on specific cognitive domains in older adults (e.g., executive functions, memory, and processing speed). These areas have been studied extensively with aging-related dysfunctions being frequently reported (van den Berg et al., 2009).

### Executive functions

Executive functions are a set of interrelated cognitive abilities for achieving goal-directed behaviors. As the frontal lobes of the brain are particularly vulnerable during aging, deficits in executive functions have been prevalent among older adults (Cowell et al., 1994). Older adults demonstrate declines in generalized inhibitory performance, irrespective to the stimuli, than their normal weight counterparts (Mobbs et al., 2011). Such executive deficits are especially apparent when older adults possess a combination of high adiposity and elevated blood pressure (Waldstein and Katzel, 2005). Reduced frontal lobe volume is normal in aging; however, obesity seems to exacerbate the decline. Diminished executive functioning in obese older adults was partly explained by reduced gray matter volume in the left orbitofrontal region compared to the non-obese controls (Walther et al., 2010), suggesting that obesity can accelerate cognitive aging (e.g., poorer executive control) in obese adults.

### Memory

Like executive control, memory is integral in functional living, and has been an important area of scientific inquiry. Obese or overweight older adults’ scored more poorly on free recall tasks than non-obese controls (Benito-León et al., 2013). A longitudinal study suggested that retention of visual information declined over time and is related to increased obesity in aging (Gunstad et al., 2010). Normal weight participants also outperformed overweight and obese peers in semantic memory tests (Nilsson and Nilsson, 2009). Memory formation and retention both involve the frontal lobes and hippocampus. In addition to frontal lobe atrophy, the temporal lobe, where the hippocampus is located, also undergoes considerable shrinkage with age (Cowell et al., 1994). Among older adults, higher visceral adiposity is inversely associated with hippocampal volume and verbal memory (Isaac et al., 2011). Obesity accelerates cognitive aging in a widespread area, spanning across a range of neural regions of the brain.

### Processing speed

Obesity can also be related to information processing speed. Obese or overweight older adults are more likely to be in the lowest quartile of a Trail Making Task (part A) and verbal fluency, a measure of processing speed in which participants are asked to say as many words per category as possible within a given period of time (Gunstad et al., 2010; Benito-León et al., 2013). Moreover, older adults with greater BMI or waist circumference and higher systolic or diastolic blood pressure show reduced ability to initiate motor responses (Waldstein and Katzel, 2005). Mechanisms underlying obesity’s effect on impeding motor initiation is unknown. It may be due to neuronal slowness in certain brain regions (e.g., motor areas) responsible for initiating bodily movements.

## Mechanisms Underlying Obesity Effect on Cognition

**Figure 1:**
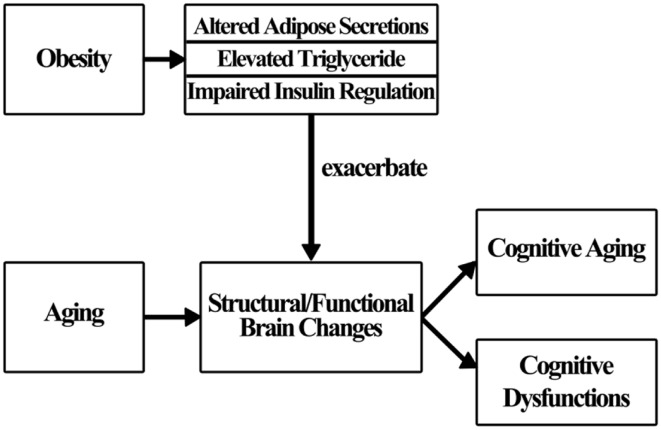
**Schematic presentation of how obesity influences cognition**.

Obesity does not influence cognition directly (Sellbom and Gunstad, 2012); rather, a number of factors are thought to mediate the effects of obesity on brain changes and cognition (Figure 1). In this section, some underlying mechanisms are discussed, though additional study is warranted for a more complete understanding.

Many theories concerning the obesity/cognition linkage have been proposed over the past several decades. Researchers suggest that cardiovascular risk factors and diseases (e.g., diabetes) associated with obesity partially mediate the effects of obesity on cognition (Elias et al., 2012). This means that the presence of obesity alone is not enough to affect cognition. Rather, the emergence of associated risk factors and diseases may play an important role in the process of impairing cognition. In the following section, three mechanisms: altered adipose tissue secretion, elevated triglyceride level, and impaired insulin regulation are examined relative to their effect on cognition (Smith et al., 2011).

Obese individuals possess more adipose tissues for fat storage. Adipose tissue produces many substances important for metabolism (adipokines) and inflammation (cytokines) (Luchsinger and Gustafson, 2009). A high level of adipokines secreted by adipose tissue can be linked to structural brain abnormalities (Sellbom and Gunstad, 2012). In addition, systemic inflammation can be a result of certain circulating factors produced by the adipose tissue (Knopman and Roberts, 2010). Leptin, an inflammatory cytokine produced by the adipose tissue, can influence neuronal excitability in the brain, and modulate inflammatory signals in microglia (Pinteaux et al., 2007; Tang et al., 2007; Diano and Horvath, 2008). Chronic elevation of leptin in obese individuals probably results in leptin resistance which is associated with cognitive deficits and inability to regulate weight (Landin et al., 1990; Chaldakov et al., 2003; Yaffe et al., 2003; Kershaw and Flier, 2004; Knopman and Roberts, 2010).

The second mechanism where obesity affects cognition concerns triglyceride (a blood lipid). Although triglyceride elevation is not consistently observed in obese people, it is believed to partly mediate the effect of obesity on cognition; obesity does not impair cognition directly. However, an indirect effect results through a heightened level of triglyceride that is obesity-related (Smith et al., 2011). High levels of triglyceride impede leptin transportation across the blood-brain barrier (Banks et al., 2004). Triglyceride adversely affects the neural system after its breakdown into free fatty acids (Bruce-Keller et al., 2009). Reduced triglyceride levels improve cerebral blood flow and performance in the Cognitive Capacity Screening Examination (Rogers et al., 1989). Nevertheless, research on triglyceride’s impact on cognition is still sparse; more studies are required to fully understand its role in influencing cognition.

Insulin resistance, which is usually highly correlated with obesity, is related to cognitive deficits (Greenwood and Winocur, 2005). With insulin resistance, cells fail to respond to insulin to metabolize glucose, triggering a further surge of insulin. The excess insulin produced increases the beta-amyloid level that has long been thought to be responsible for the development of AD (Craft, 2005). Moreover, there are numerous insulin-sensitive glucose transporters in the medial temporal region serving memory functions. Insulin resistance may significantly affect memory performance (Watson and Craft, 2003). However, debates and controversies about the above described mechanisms described are still ongoing. More research is necessary to fully understand the mechanisms. Figure 1 shows how obesity may influence cognition.

## Exercising and its Benefits on Obesity and Cognition

Exercise improves chronic disease, reduces functional limitations, and delays cognitive impairments. Furthermore, exercise improves cognitive aging by influencing cognition directly, or indirectly, by reducing obesity (Nelson et al., 2007). We summarize the efficacy of different types of exercise on obesity and cognitive improvements in older adults.

### Obesity

Sedentarism, leading an inactive lifestyle, is strongly related to the occurrence of obesity while physical activity level is negatively associated with pericardial fat and BMI (Hamer et al., 2012; Xue et al., 2012). Compared with normal weight counterparts, obese older adults are less active. This increases the tendency to become obese, further decreases one’s activity level (Jenkins and Fultz, 2008), and creates a vicious cycle. Mortality related risks increase significantly as obesity increases (Woo et al., 2013). Therefore, introducing older adults to a more active lifestyle is a key to reducing obesity. Unlike weight management goals for younger adults, who may want to lose weight aggressively, the goal for older adults should be to stabilize weight while avoiding further weight gains (Chau et al., 2008). According to Shiroma et al. (2012), moderate intensity exercise for 1 h/day is effective in preventing weight gains in obese older adults while yielding an array of social, physical, and cognitive benefits. Exercise helps prevent increases in fat weight while preserving, or even increasing, lean body (muscle) mass in older adults. This is important for balance maintenance and fall prevention (Stehr and von Lengerke, 2012). Further, a recent finding indicated a malfunction in fatty acid oxidation in skeletal muscle among obese individuals. This can be modulated by exercise (Battaglia et al., 2012). Clearly, on the basis of these studies, exercise can be beneficial to older adults, especially those who are obese.

Aerobic exercise has also shown significant positive effects for younger adults. Aerobic exercise has similar or more benefits for older adults in combating obesity or its related disorders. When older adults engage in more aerobic exercise, fat mass decreases, fat-free mass increases (Jackson et al., 2012). Moderate- or vigorous-intensity aerobic exercise 3 days a week for 20 weeks led to a dramatic reduction of 25% abdominal visceral fat among post-menopausal women aged 50- to 70-years-old (Nicklas et al., 2009). Aerobic exercise five times a week resulted in lower obesity indices, including body weight, BMI, waist circumference, and percentage body fat (Foster-Schubert et al., 2012). Anaerobic exercise also demonstrated favorable outcomes. Resistance training increased muscle strength and decreased arterial pressure (Figueroa et al., 2013). Moreover, it reduced fat-free mass loss that is prevalent among older adults (Frimel et al., 2008). The beneficial effect of anaerobic training has been observed even in cognitively impaired older adults (Heyn et al., 2008).

Both aerobic and anaerobic exercise should be included in a training regimen targeting older adults. A 6-week comprehensive exercise program led to increases in aerobic fitness, muscle fitness, lean mass, high density lipoprotein cholesterol, and decreases in abdominal fat (Stewart et al., 2005). Moderate physical activity, including aerobic and anaerobic components over a year, improved physical functions in healthy older adults. However, the benefits were less in obese participants. Furthermore, there were reductions in waist circumference for both obese and non-obese older adults over time within the intervention period (Manini et al., 2009). The results suggested that a training program with both aerobic and anaerobic components is most effective in healthy older adults. In a study comparing the efficacy of aerobic and anaerobic trainings administered alone and together, aerobic training alone was sufficient for reducing fat mass and body weight. However, inclusion of resistance exercise to a training program was important for increasing lean mass in obese individuals (Willis et al., 2012). Thus, a combination of aerobic and anaerobic training is ideal for older obese adults. Following improvements in obesity, the associated adverse effects to cognitive aging can be reduced.

In addition to the differential effects of aerobic and anaerobic exercises on physical functions, these two types of exercise also generated differential impacts on the brain. Aerobic exercise leads to an increase in brain volume and gray/white matter while anaerobic exercise does not (Colcombe et al., 2006). However, aerobic and anaerobic exercises lead to increased connectivity in non-task-dependent and task-dependent neural networks respectively (Voss et al., 2010). Thus, a combination of aerobic and anaerobic training is ideal, even crucial, for older obese adults, and the exercise does not need to be vigorous to be beneficial. Exercise programs (aerobic and anaerobic trainings 2–3 days a week) lasting for 3–12 months can yield an array of significant benefits (Vincent et al., 2012; Xue et al., 2012).

### Cognition

Exercise and physical activity help maintain and revitalize cognition in older adults. By definition, physical activity is “bodily movement produced by skeletal muscles resulting in energy expenditure” whereas exercise refers to physical activity that is “planned, structured, and repetitive and has as a final or an intermediate objective the improvement or maintenance of physical fitness” (Caspersen et al., 1985, pp. 126–128). Physical activity and exercise are associated with improvements in global cognitive measures and a range of specific cognitive abilities (e.g., attention, information processing speed, executive functions, memory; Eggermont et al., 2009; Eskes et al., 2010; Flöel et al., 2010; Rosano et al., 2010; Wilbur et al., 2012; Benedict et al., 2013).

Sufficient physical activity and exercise also help maintain brain health by delaying cognitive declines (Weuve et al., 2004; Kramer et al., 2005; Kramer and Erickson, 2007; Sofi et al., 2011). Physical activity protects older adults against AD, cognitive impairments, and dementia (Laurin et al., 2001). Physically active older adults were 21% less likely to have dementia compared to inactive controls (Bowen, 2012). At a 10-year follow up, exercise quantity was inversely related to the onset of cognitive impairments (Jedrziewski et al., 2010). Exercise and physical activity are advised for healthy individuals and, for many reasons, can be very effective among older adults who are physically frail or cognitively impaired (Graff-Radford, 2011; Langlois et al., 2013).

The effects of aerobic exercise on cognition have been studied extensively. The most frequently reported benefits are improved memory and executive functions (Albinet et al., 2010; Erickson et al., 2011; Voelcker-Rehage et al., 2011). Aerobic fitness can uniquely account for volume variance of hippocampus which plays a significant role in memory and is vulnerable to obesity-related declines (Bugg et al., 2012). Neuroimaging studies revealed that aerobic exercise increased hippocampal volume and functional connectivity among brain areas (Voss et al., 2010; Erickson et al., 2011). Thus, aerobic exercise can, presumably, benefit memory functions by protecting the hippocampus from shrinkage and improving its connectivity with peripheral regions.

Obesity is a major risk factor of diabetes. Similar to obese individuals, diabetics demonstrated cognitive deficits including memory declines (Ryan and Geckle, 2000; Debling et al., 2006). Exercise has been shown to improve memory functions in people who are obese and/or diabetics. Compared to the control group (1-h stretching for three times a week), aerobic exercisers (1-h walking or jogging for three times a week) demonstrated better memory recall after 6 months of training (Watson et al., 2006). Besides memory functions, studies also showed faster processing speed and reduced risk of vascular dementia in older aerobic exercisers (Ravaglia et al., 2008; Maki et al., 2012). In addition, aerobic fitness was positively related to white matter integrity in the prefrontal regions (Marks et al., 2007).

Research has also demonstrated positive outcomes from anaerobic exercise in relationship to cognition. Stretching exercises improved global cognitive functions and memory (Lam et al., 2011). Moderate and vigorous-intensity resistance training heightened global cognitive functions, executive functions, and memory (Lachman et al., 2006; Cassilhas et al., 2007; Anderson-Hanley et al., 2010). Tai Chi was also effective for cognition as older adults in the exercise group increased brain volume and outperformed those without intervention in a dementia rating, a Trail Making Test, auditory verbal learning, and verbal fluency after 40 weeks (Mortimer et al., 2012). Though a number of studies show positive results from anaerobic exercise, not all anaerobic exercises are beneficial. Yoga, for example, has not been found to positively affect older adults’ cognitive abilities (Oken et al., 2006). The reason underlying the null effect of yoga on cognition is unclear; it may be a result of short exercise time (a 90-min session a week) and/or relatively low training intensity in the yoga program.

Physical activity generally brings favorable changes (neurodegenerative, neuroprotective) to the neural system (Dishman et al., 2006). At the cellular level, physical activity stimulates synaptic and neuronal plasticity, mostly through neurogenesis (formation and development of new cells induced by neurotrophic factors, such as BDNF), and protects against neuronal damage and neurotoxins (Vaynman and Gomez-Pinilla, 2005; Dishman et al., 2006; Archer, 2011; Ruscheweyh et al., 2011). Physiologically, physical activity is related to fitness that has been associated with increased hippocampal volume, improved neural connectivity, less gray/white matter loss in healthy older adults, and reduced brain atrophy in AD patients (Erickson et al., 2009; Burdette et al., 2010; Ahlskog et al., 2011; Gow et al., 2012). Empirical evidence suggests that both aerobic and anaerobic exercises are important in maintaining cognitive and brain vitality, especially for older adults (Voss et al., 2011).

## Implications and Future Research

A large body of research suggests that exercise is an effective way to reduce obesity, enhance cognition, and slow cognitive declines in older adults. Clearly, older adults need exercise regularly to achieve optimal body weight and brain health. However, when will exercise exert its greatest influence in reducing obesity and cognitive aging in older adults? Most studies indicate that midlife (approximately 40- to 60-year-old) is the critical period for optimal benefits of exercise in the attenuation of cognitive aging. To fully benefit from exercise regimens in reducing the impacts of cognitive aging, one should start exercise by 40–60 years of age. Midlife obesity has detrimental effects on cognition in older adults. Middle aged adults with high BMI experience accelerated cognitive declines (Dahl et al., 2010). Compared to normal weight people, overweight/obese middle adults have shown poorer performance in a variety of cognitive abilities, from global cognition, perceptual speed, executive functions, and memory in later life (Sabia et al., 2009; Hassing et al., 2010; Lee et al., 2010; Debette et al., 2011; Dahl et al., 2013).

Additionally, BMI has been inversely associated with brain volume in middle aged adults (Ward et al., 2005). High midlife BMI is related to abnormalities in neurons and myelin sheath, especially in the frontal lobe. This suggests a higher risk of developing neurodegenerative diseases in obese middle aged adults when they become older (e.g., AD; Gazdzinski et al., 2008). Thus, exercising by midlife is of utmost importance in preventing obesity and slowing the cognitive downturn in late life. Exercise helps maintain most aspects of fitness, but also aids in the delay of neurodegenerative diseases, as the risks of developing dementia and MCI are negatively related to exercise during midlife (Ahlskog et al., 2011). As indicated by the research discussed, it would be best to start exercise at or before midlife as a means to improve obesity and counteract cognitive aging in later life, otherwise, the benefits of exercise will be compromised.

Exercise clearly has positive impacts in reducing obesity, cognitive aging, and related dysfunctions. However, several related issues remain unclear and warrant further examination. First, the exact mechanism of obesity’s effects on cognition is still unknown. Future research may benefit from technological advances that enable testing existing and future theories on humans and animals. Second, the search for optimal exercise parameters related to older adulthood, obesity, and cognition is ongoing. Current consensus suggests that both aerobic and anaerobic exercise should be included. But the optimal amount and intensity of each type of exercise are still in question. More randomized control trials are needed in this area. Third, studies have demonstrated that there are interactions among obesity indices on cognition. In Kerwin et al.’s (2010) study, in which only post-menopausal women were recruited, there was an interaction between BMI and waist-hip ratio on cognition. This may have contributed to a reduction in cognitive function. This was only observed when both high BMI and small waist-to-hip ratio were present.

In another study, the association between global cognitive score and BMI was significant only when abdominal obesity was present (Jeong et al., 2005). This observation suggested that influences of different obesity indices on cognition were inconsistent. Investigations into the aggregate and separate effects of individual obesity indices on cognitive abilities could be helpful in explaining the obesity-cognition relationship. Finally, some cognitive advantages exist among older adults who are obese over those who have normal weight (Nilsson and Nilsson, 2009). However, after dividing older adults into young-old (≤70-year-old) and old-old (≥70-year-old) groups, obesity (particularly in those with high visceral adiposity) was associated with poorer cognition in young-old adults only, but not in the old-old adults (Yoon et al., 2012). The term “obesity paradox” has been used to describe this phenomenon, with the underlying explanations still being unclear.

## Conclusion

Obesity in older adulthood affects health and cognition. As a result of increasing obesity in the American population, and the increase in number of older Americans, this phenomenon has become a persistent and pressing human, societal, and scientific issue. Obesity exacerbates age-related declines in a range of cognitive abilities (e.g., executive function, memory, and processing speed) by altering adipose secretions, elevating triglyceride level, and impairing insulin regulation to affect structural and functional brain changes in the aging process. Over the past few decades, evidence suggests there are many positive effects of exercise for decreasing obesity and improving cognition. Aerobic exercise reduces fat mass and weight. Anaerobic exercise is crucial for increasing lean body mass that is important in supporting physical functions and preventing injuries in older adults. Despite conflicting results about optimal training parameters (e.g., type, frequency, duration), exercise comprising both aerobic and anaerobic components is generally recommended for obese older adults as a means of improving physical health while ameliorating cognitive declines. Exercise should be promoted in older adults as a cost-effective, efficient, and viable way to reduce obesity and cognitive dysfunctions.

## Conflict of Interest Statement

The authors declare that the research was conducted in the absence of any commercial or financial relationships that could be construed as a potential conflict of interest.
